# Case report: Secondary peristomal cutaneous squamous cell carcinoma following multimodal treatment for rectal cancer

**DOI:** 10.1016/j.ijscr.2025.112112

**Published:** 2025-10-26

**Authors:** Elena Knochenhauer, Andre Schreiber, Anne Glitsch, Stephan Kersting

**Affiliations:** a Department of Surgery, Universitätsmedizin Greifswald, Ferdinand-Sauerbruch-Straße, 17475, Greifswald, Germany

**Keywords:** Rectal carcinoma, Squamous cell carcinoma, Tumour aftercare, Complication management, Peristomal carcinoma

## Abstract

**Introduction:**

This case report describes the rare development of a highly differentiated, keratinising squamous cell carcinoma on the skin of the anus praeter following multimodal therapy for rectal cancer. The case was successfully managed through structured follow-up care and interdisciplinary collaboration.

**Case presentation:**

We report on the complex case of a patient with rectal carcinoma, which was treated with neoadjuvant radiochemotherapy, followed by surgical resection and adjuvant chemotherapy. During treatment, a low anterior resection syndrome developed, resulting in the creation of an end colostomy. Verrucous skin ulcerations developed around the stoma, leading to the development of squamous cell carcinoma of the skin based on this chronic inflammation.

**Discussion:**

Secondary malignancies are a significant late complication of radiotherapy or combined radiochemotherapy. While high doses of radiation cause cell death, malignant transformations can be promoted in the low-dose peripheral areas of the radiation fields. In the present case, however, the chronic inflammatory skin change indicates inflammation-induced carcinogenesis, which is described in the literature as a central pathogenetic mechanism.

**Conclusion:**

This case highlights the importance of close follow-up care and a holistic approach to patient care. It also illustrates the importance of recognising and managing treatment complications. Furthermore, it raises awareness of the development of secondary malignancies and malignant transformations.

## Highlights

• Rectal carcinoma
• Tumour aftercare
• Secondary malignancy
• Complication management
• Peristomal carcinoma

## 1 Introduction

Secondary malignancies are a rare but serious complication in patients with previous tumour therapy. For example, radiotherapy leads to a small but significant increase in the development of secondary tumors [1]. In rectal cancer patients in particular, the cumulative incidence of secondary neoplasia can be approximately doubled compared to an unirradiated group [2]. In the present case, we report on the development of a highly differentiated, keratinising squamous cell carcinoma due to chronic inflammation of the adjacent skin of the anus praeter. The patient initially underwent neoadjuvant, surgical and adjuvant treatment for rectal carcinoma. The case shows that chronic inflammation, including in a stoma, can be a risk factor for malignant transformation [3]. There is a higher risk of transformation, particularly in the case of long-term existence and progressive irritation [4].

The possible occurrence of secondary malignancies emphasises the need for close, structured follow-up care. The patient was comprehensively informed about the course of the disease and the treatment received and consented to the publication of the case data. The work has been reported in line with the SCARE criteria [5].

## 2 Case presentation

The patient, who was 50 years old at the time of the initial diagnosis, reported a history of intermittent blood deposits in the faeces. A colonoscopy revealed a suspected rectal carcinoma at a height of 13 cm from the anocutaneous line, which was confirmed histologically after sampling. The subsequent staging revealed an initial tumour stage of uT3 N+, whereupon the interdisciplinary tumour board recommended neoadjuvant radiochemotherapy according to the Erlanger protocol in 2015, which the patient received between June and July 2015 with 5-fluorouracil.

The rectal carcinoma was then surgically treated in September 2015 by a low anterior rectal resection with a protective ileostomy. Histopathological examination revealed adenocarcinoma of the rectum with a postoperative tumour stage of ypT3 ypN0(0/20) cM0 L0 V0 R0 G2, which corresponds to stage IIa according to the UICC (7th edition, 2010). As part of the CoCStom study [6], the ileostomy was repositioned early on postoperative day 8. Due to anastomotic insufficiency, a new ileostomy was created in October 2015. Between November 2015 and February 2016, the patient received adjuvant chemotherapy to complete the Erlanger protocol.

The protective ileostomy was then repositioned. In the further course, the patient developed low anterior resection syndrome (38 points), which manifested itself in pronounced faecal incontinence. An electrode for sacral nerve stimulation was implanted for therapeutic purposes. However, this did not have the desired effect, so a terminal descendostomy was created at the patient's request in June 2017 due to persistent faecal disorders. After successful colostomy placement, the patient reported a significant increase in quality of life due to the controllable stool regulation. At the same time, the inserted nerve stimulator was explanted.

**Fig. 1:**
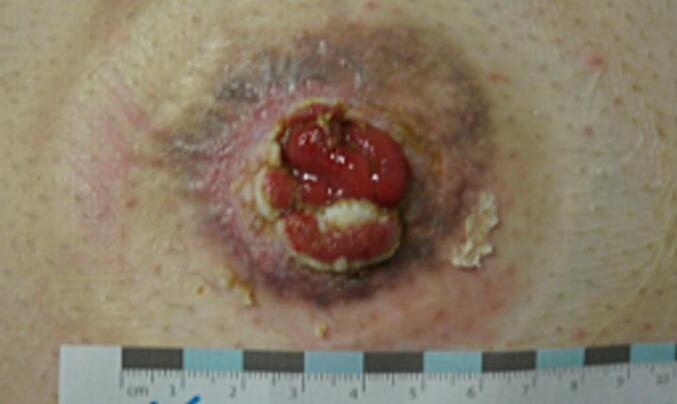
Initial perifocal skin lacerations around the stoma.

The structured tumour follow-up of the rectal carcinoma was completed in September 2021 when the patient was tumour-free. From around this time, the patient reported painful skin irritation. The physical examination revealed verrucous skin changes at the edge of the stoma with perifocal skin lacerations (Fig. 1).

First, careful local wound care was initiated. The stoma care was checked and adjusted by a stoma therapist, stoma powder and skin protection were used to reduce maceration and protect the peristomal skin.

**Fig. 2:**
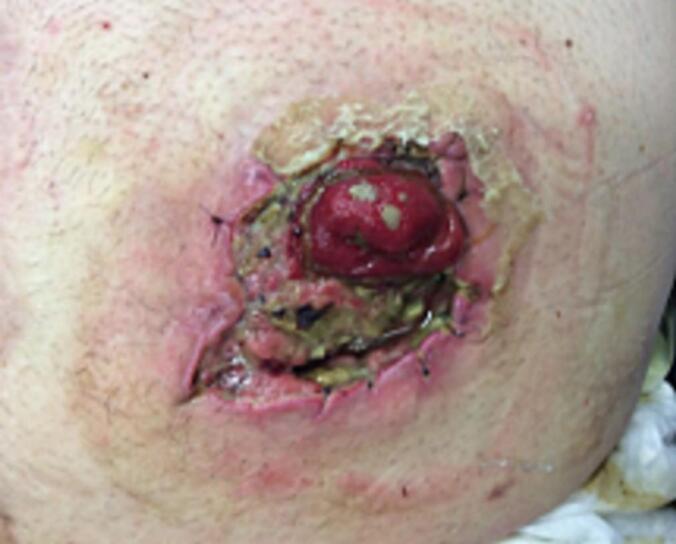
Increasing skin lacerations, enlarged in diameter and with clear inflammation.

**Fig. 3:**
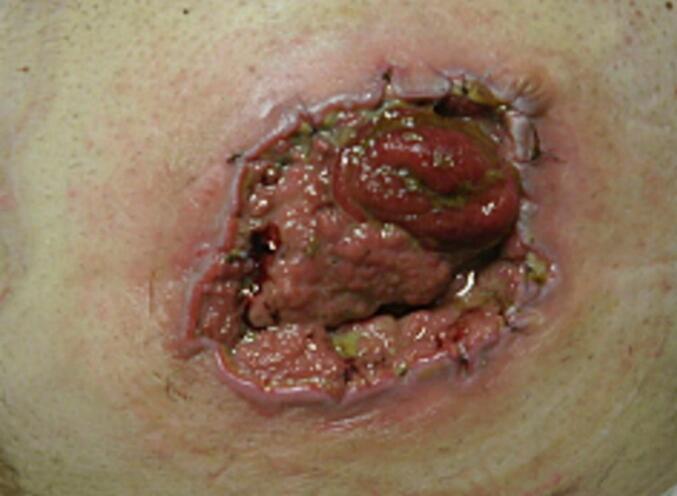
Increasing skin lacerations, enlarged in diameter and with clear inflammation.

As the patient wanted to take care of the wound himself, he was trained to change the dressing regularly under sterile conditions. Further wound checks were carried out by the family doctor and at our surgery every three to six months. In May 2022, these cutaneous changes increased, the diameter increased and there was also clear inflammation of the tissue. The affected area was rinsed with saline solution, and wound swab was taken for microbiology testing to rule out infection. The patient was pain-compensated at this time. In the absence of clinical symptoms at this point, it was agreed with the patient to continue wound care and wound checks (Fig. 2, Fig. 3).

**Fig. 4:**
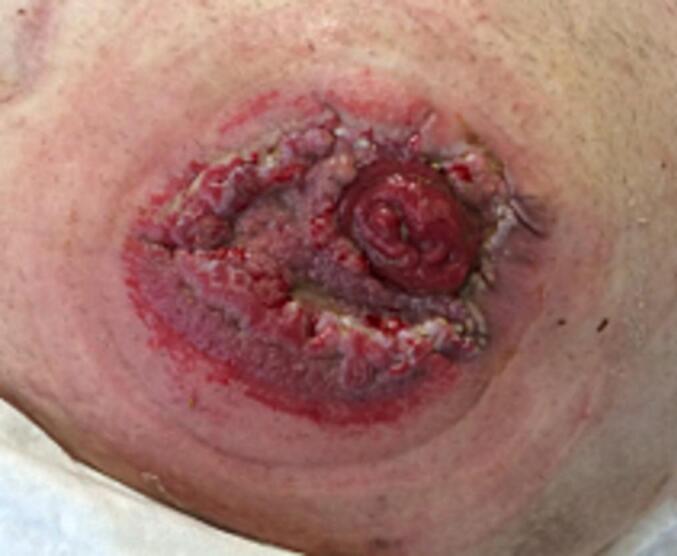
Size-progressive, painful skin laterations - preoperative photo documentation.

The diameter of the skin lesions continued to increase. However, the patient was now clearly dissatisfied with the pain. He reported increasingly persistent pain in the peristomal skin area and a burning sensation, especially when changing the stoma appliance. In addition, bleeding from the affected skin area was observed (Fig. 4). Due to the non-healing ulcer, the symptoms and the abnormal growth, a malignant cause of the skin findings could not be ruled out with certainty. In the further course, there was a persistent reduction in the amount of stool passing through the stoma, accompanied by a change in stool consistency. In addition, increasing abdominal discomfort and occasional nausea occurred. Digital examination during the physical examination confirmed the presence of stoma stenosis. Based on the findings, surgical revision was indicated (Fig. 4).

In the subsequent surgical revision of the stoma, a highly differentiated, keratinising squamous cell carcinoma was diagnosed histopathological at the transition of the anus praeter naturalis to the skin due to chronic inflammation.

The result of the histopathological examination revealed a carcinoma that surrounded the stoma like a seam, with a width of about 1.5 cm, and focally reached the edge of the incision at skin level. The initial tumour stage was determined as pT2 L0 V0 R1 cM0 malignancy grade: G1. After staging was completed and a further local surgical resection was performed due to an initial R1 situation, the carcinoma was resected in a healthy state. The final postoperative tumour stage was pT3 L0 V0 R0 cM0 malignancy grade: G1, with an overall diameter of 5.3 cm.

Today, the patient is 64 years old. At the current follow-up, the patient is tumour-free, symptom-free and enjoys a high quality of life. The descending ostomy is pumping properly and the skin surrounding the stoma is free of irritation.

**Fig. 5:**
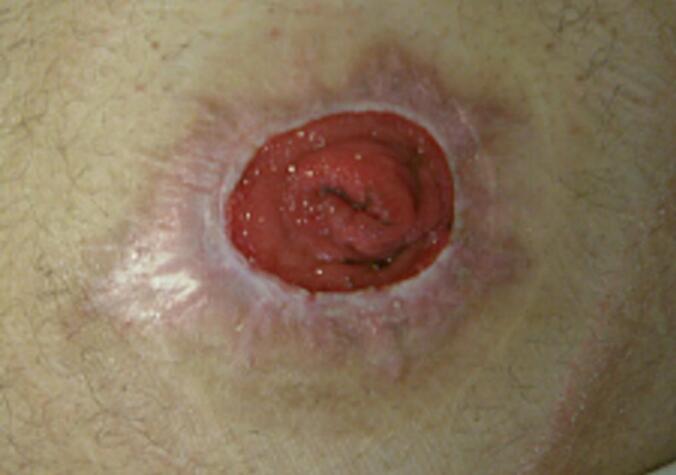
Current follow-up 2 years postoperatively.

The last endoscopy of the rectal stump was performed in April of this year and showed no evidence of a recurrence of the rectal carcinoma (Fig. 5).

## 3 Discussion

The origin of the malignant peristomal skin lesion may be metastasis, metachronous carcinomas or purely isolated secondary tumors. Metachronous colorectal carcinomas occur in 5–10 % of cases of colorectal carcinomas [7]. Metastatic diseases typically occur in the first 2–4 years after surgery [7]. In the present case, the late manifestation, the cutaneous localisation and the different histology compared to the original rectal adenocarcinoma suggest an isolated secondary squamous cell carcinoma of the stoma margin. Similar cases of peristomal squamous cell carcinoma were described by Hani et al. (2020) in a literature review on ileostomies, in which chronic inflammation was also highlighted discussed as a predisposing factor for tumour development [8].

Secondary malignancies are a significant late complication of oncological therapies and particularly affect patients who have undergone radiotherapy or combined radiochemotherapy. The literature shows that any genotoxic therapy can trigger secondary malignancies after a latency period of several years to decades [1]. The incidence of such tumors depends on various factors. Ionizing radiation induces genomic changes that lead to cell death in high-dose volumes, while in low-dose ranges they can promote malignant transformation with tumour formation potential. This dose dependency explains why secondary tumors typically develop at the edges of the irradiation fields [9,10].

In the present case, the patient received neoadjuvant radiochemotherapy according to the Erlanger protocol to treat a rectal carcinoma, which led to successful control of the primary tumour. However, seven years after the initial diagnosis, a secondary squamous cell carcinoma of the skin appeared at the stoma margin. This latency period is consistent with the known delay in the development of secondary tumors.

Birgisson et al. reported that in patients with resected rectal cancer, the cumulative incidence of secondary tumors in organs within or adjacent to the irradiated region was approximately twice as high in the irradiated group as in the non-irradiated group. In our case, however, the stoma is not adjacent to the irradiated area, which makes radiation-induced tumour development unlikely [11].

An alternative pathophysiological basis for the development of secondary tumors after radiotherapy is the induction of chronic proliferative processes as a long-term consequence of the therapy [1].

Chronic inflammation is considered a major risk factor for malignant transformation. Coussens et al. and Balkwill et al. described chronic inflammation as a driving force of carcinogenesis based on the following mechanisms [3,4]: Chronic inflammation creates a microenvironment that supports tumour cells by releasing growth factors, cytokines and enzymes. This promotes cell proliferation [3]. In addition, various immune cells such as macrophages, neutrophils and mast cells infiltrate the tumour tissue and influence the developing tumour by secreting pro-inflammatory mediators. The immune response is thus modulated by the chronic inflammation [3].

Ultimately, the inflammatory mediators can promote DNA damage, which suppresses apoptosis and stimulates angiogenesis. All in all, this promotes tumour growth [3].

In the present case, verrucous, inflammatory skin changes were present on the skin of the stoma edge over a long period of time. This chronic inflammatory process was always progressive. Finally, a highly differentiated squamous cell carcinoma of the skin developed based on this chronic inflammation.

The case illustrates the need for structured tumour aftercare, which focuses not only on recurrences of the primary tumour, but also on the development of possible secondary neoplasms after a latency period. Comprehensive care, which includes interdisciplinary examinations in addition to oncological expertise, appears to make sense to be able to recognize inflammatory changes and rule out malignant transformation at an early stage, for example. This could reduce the risk of overlooked secondary tumors.

Future research could characterize specific risk factors for secondary malignancies more precisely and develop individual follow-up protocols. In particular, the role of the immune response to chronic inflammation and its influence on carcinogenesis could be further investigated in the future.

## 4 Conclusion

The complex case of this patient with histologically confirmed squamous cell carcinoma following multimodal treatment for rectal carcinoma illustrates the relevance of close tumour follow-up. The case raises awareness of the importance of recognising and managing treatment complications and preventing secondary malignancies. It impressively shows that unexpected complications can occur even with guideline-compliant therapies, which requires interdisciplinary care and individual adaptation of structured tumour aftercare.

## Author contribution

Operation procedure AS, AG; original draft writing EK; review and editing AS, SK; supervision SK

## Consent for publication

Written informed consent was obtained from the patient for publication of this case report and accompanying images. A copy of the written consent is available for review by the Editor-in-Chief of this journal on request.

## Ethical approval

Not applicable.

## Guarantor

Elena Knochenhauer

## Registration of research studies

N/A

## Sources of funding

Not applicable.

## Declaration of competing interest

The authors have no competing interests.
